# Early memories of warmth and safeness with peers and borderline personality features in adolescents: The mediator role of self-disgust

**DOI:** 10.1192/j.eurpsy.2023.423

**Published:** 2023-07-19

**Authors:** C. Pinto-Gouveia, A. Rocheteau, D. Carreiras, M. Cunha

**Affiliations:** ^1^Psychiatry, CENTRO HOSPITALAR E UNIVERSITÁRIO DE COIMBRA; ^2^Instituto Superior Miguel Torga; ^3^Center for Research in Neuropsychology and Cognitive and Behavioral Intervention, University of Coimbra, Coimbra, Portugal

## Abstract

**Introduction:**

Adolescence is a development stage that stands out by normative challenges that comprise the creation of representations about the self and the others, the definition of a sense of identity, and adaptation of emotional regulatory strategies and behaviors in social contexts, particularly within the group of peers. However, these developmental tasks can raise psychological difficulties that may facilitate the emergence of psychopathology in adolescents.

**Objectives:**

Analyze the role of self-disgust in the relationship between early memories of warmth and safeness with peers and borderline personality features in adolescents.

**Methods:**

Sample was composed of 451 adolescents (260 females and 185 males), with a mean of 15.55 years of age (SD = 1.49), who completed self-report questionnaires to assess early memories of warmth and safeness with peers (EMWSS_Peers_-A), self-disgust (MSDS-A) and borderline features (BPFS-C). Pearson’s correlation coefficients and a path analysis were performed using SPSS and PROCESS Macro.

**Results:**

Girls had higher values of self-disgust and borderline features than boys, as well as similar levels in early memories of warmth and safeness with peers. The mediation model was significant and explained 54% of the variance of the borderline traits, with early emotional positive memories with peers and self-disgust dimensions contributing significantly to the explanation model. Gender and exclusion (a specific dimension of the self-disgust scale) did not have predictive power in borderline features.

**Conclusions:**

Our results contribute to a better understanding of the psychological mechanisms involved in the development of borderline traits and consequently to clinical practices and research on adolescence.

**Disclosure of Interest:**

None Declared

